# An Efficient, Clinically-Natural Electronic Medical Record System that Produces Computable Data

**DOI:** 10.5334/egems.202

**Published:** 2017-12-15

**Authors:** Brent C. James, David P. Edwards, Alan F. James, Richard L. Bradshaw, Keith S. White, Chris Wood, Stan Huff

**Affiliations:** 1 Intermountain Healthcare, US

**Keywords:** natural language processing, next generation electronic medical records, clinical decision support

## Abstract

Current commercially-available electronic medical record systems produce mainly text-based information focused on financial and regulatory performance. We combined an existing method for organizing complex computer systems—which we label activity-based design—with a proven approach for integrating clinical decision support into front-line care delivery—Care Process Models. The clinical decision support approach increased the structure of textual clinical documentation, to the point where established methods for converting text into computable data (natural language processing) worked efficiently. In a simple trial involving radiology reports for examinations performed to rule out pneumonia, more than 98 percent of all documentation generated was captured as computable data. Use cases across a broad range of other physician, nursing, and physical therapy clinical applications subjectively show similar effects. The resulting system is clinically natural, puts clinicians in direct, rapid control of clinical content without information technology intermediaries, and can generate complete clinical documentation. It supports embedded secondary functions such as the generation of granular activity-based costing data, and embedded generation of clinical coding (e.g., CPT, ICD-10 or SNOMED). Most important, widely-available computable data has the potential to greatly improve care delivery management and outcomes.

## Introduction

Over the past 60 years, digital automation has massively improved business operations and human life. Such development in health care delivery has advanced only to “digital paper:” While some clinical information is captured in a fully computable format that can support digital automation (e.g., laboratory results), most still takes the form of text records. Natural language processing (NLP) software can successfully convert highly-structured textual records into computable data, but still faces significant challenges when confronted by unstructured clinical text [8]. This has greatly limited the potential contributions that automation can offer to optimal clinical practice.

The amount of unstructured text captured in electronic medical records (EMRs) has grown rapidly. Financial operations and regulation drive much of that clinical documentation increase [1]. Clinicians use a combination of direct data entry, free-text entry, voice recognition, transcribed dictation, and “cut and paste” to provide justification for billing and clinical quality oversight codes. Sinsky et al. found that physicians spend 2 hours doing EMR-based clerical work for every hour they spend in direct patient contact [2]. Electronic documentation is the leading single cause of increasing rates of physician burnout [1]. Onto that primarily text-based, financial-oriented documentation chassis, EMR systems “bolt on” clinical decision support (CDS).

What form might an EMR take if it were designed primarily for CDS? What proportion of all information could such a system capture as computable data, allowing better digital automation of clinical care processes?

## Methods

### Designing an EMR system to prioritize and optimize clinical decision support

In 1991 we discovered a strong method for building CDS into clinical practice. Care Process Models (CPMs) deploy evidence-based best practice guidelines into clinical workflows as “shared baseline” protocols [3]. They are a form of Lean “mass customization” [4]. CPMs demand that clinicians adapt “standard work”—the CPM, built into clinical workflow—to the specific, unique needs of each individual patient. Care delivery organizations have successfully used CPMs to deploy appropriate use criteria (indications for referral, testing or treatment), diagnostic support (with embedded clinical data capture and recommended imaging and testing orders), standard treatment protocols (such as standing order sets), and documentation.

CPMs are based on Deming’s process management theory [5]. Processes demonstrate a hierarchical structure that starts with a set of clinical and cost end outcomes, then work through a series of intermediate outcomes (process steps), down to the level of frontline clinical workflows. Within process theory, frontline workflows represent “decision level”—the point at which patients and their clinical advisors make choices and take actions [15,16]. At decision level, a CPM becomes a set of granular, interconnected subprocesses, or “activities.” Activities are fractal: An activity can combine other activities within its own structure. Activities can also launch other activities, that operate in series or in parallel, either as “fire and forget” independent activities (asynchronous), or daughter activities that communicate with and receive direct control from their parent activity (synchronous). An ABD must support “adaptive and cooperative” workflows [7], two properties essential for clinical care delivery that are usually missing from business workflow engines. Despite a similar name, our use of the term “activity-based design” differs fundamentally from “activity centered design,” a common tool in the general medical informatics literature [11]. Activity centered design is a method for designing user interfaces based on careful analysis of the behaviors displayed by users as they interact with a technology. Our definition of ABD hews more closely to formal computer programming languages and related compiler technology.

**Figure 1:**
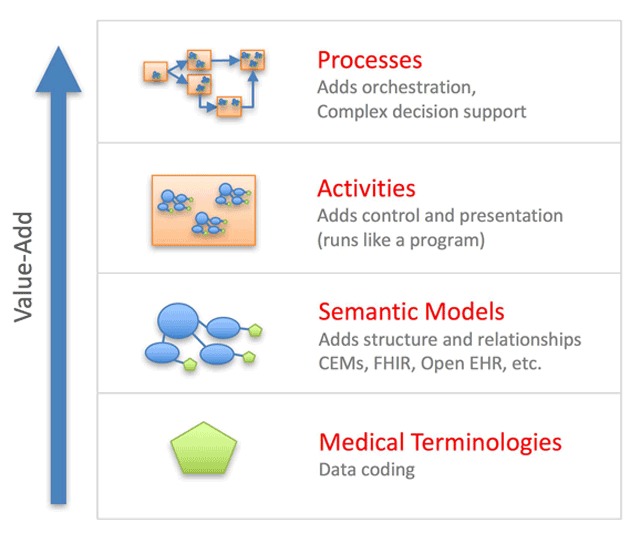
Complex processes of care can be represented in computable form using a hierarchical framework built upon a relatively simple foundation. The most basic element is a single medical concept defined within a standard medical terminology (e.g., body temperature). Multiple concepts can aggregate together into semantic models, which have sufficient complexity to represent real world data (e.g., blood pressure measurement that includes systolic blood pressure, diastolic blood pressure, the location of measurement, and the method of measurement). Semantic models, combined with control logic (e.g., default values, required vs optional data capture, dependencies, and cardinality), can be further combined to represent basic activities. Finally, activities can link together into care delivery processes (e.g., assessment and management of suspected community acquired pneumonia). The final process layer includes clinical workflows, that coordinate modular activities to orchestrate coherent care delivery. Computable clinical documentation emerges as a byproduct of the care delivery workflow.

Each activity includes a set of data elements, and provides a strong contextual framework for those data elements. We were able to map Huff’s representation of semantic clinical knowledge and content, Clinical Element Models (CEMs) [9], seamlessly to the data elements associated with activities (see Figure 1). ABD builds both clinical decision support and documentation into the workflows that define clinical care delivery, as providers interact with patients. The strong contextual framework provided by activities appears to greatly expand the ability of NLP to translate clinical text into computable data.

We built prototype EMR software to test these concepts. An Activity Composition Tool (ACT) allows a clinician, with limited training, to create activities and to define relationships among activities within an overarching CDS framework. Our aim was to place the creation of clinical content directly into the hands of clinicians, minimizing reliance on health information technology professionals – a limited resource that often becomes a bottleneck to EMR development, error fixes, and updates. The ACT allows an author to define the data elements associated with the activity, and it provides a graphical user interface so that the author can create end-user screens. The ACT uses compiler technology to guarantee that all activities conform to a rigorous computability standard: If an activity compiles cleanly, it will run within the ABD-based EMR system without breaking other parts of the system.

A prototype Activity Runtime Tool (ART) displays and executes compiled activities within care delivery workflows. We designed the ART to use existing EMR software as an underlying services-oriented architecture (SOA), to supply such functions as patient identification, data persistence, security, and order processing [12]. While the ART uses standard-based interfaces for its SOA communications (FHIR – Fast Healthcare Interoperability Resources) [10], current commercial EMRs offer limited implementations of such standards. We therefore built EMR-specific interfaces, for use until standards-based interfaces become more widely available.

Our prototype ABD system uses 3 methods to capture clinical documentation: Templates, voice recognition, and direct text entry. A template organizes the data elements associated with an activity into a user-facing data entry form. It preloads the form with a patient’s existing data, or with default values representing high probability responses. Clinical end users can “tap/click to verify” correct data elements already contained in the template, or modify them to reflect specific findings within a clinical encounter – an efficient form of “documentation by exception,” but where the clinical end user explicitly confirms all data elements captured. Voice recognition converts dictation into text, then applies NLP to convert that text into CEMs at the time of initial documentation. The resulting semantic data (CEMs) populate the associated template. Direct data entry extends beyond typing new data into a template field, or selecting among choices that a template field offers. CPMs explicitly recognize that, “with rare exception, it is impossible to create a guideline that perfectly fits any patient [6].” Clinicians may need to supplement an activity’s structured data with free text notes. The ART supports direct typing or voice recognition for that purpose.

Our ABD displays clinical documentation in 3 interchangeable formats. Most commonly, it can display patient information as a structured template, as described above. Alternatively, it can transform computable data into text, mimicking traditional documentation (“legal text”). Finally, for that portion of the data that is computable, it can identify outlier findings and present them as a “short summary.” We placed control of outlier designations into the hands of clinical end users by allowing them to assign, or modify automatically generated, levels of clinical significance. Liou has shown that it might be possible to merge templates and text into a combined user interface [17], so that end users are able to directly manipulate computable data embedded within a text-based document.

### Testing the initial activity-based design

To test our initial ABD prototypes, we recruited an experienced practicing radiologist to build a clinical application for documenting findings on standard 2-view chest radiographs performed to evaluate possible pneumonia. We tracked the time required for the radiologist to design the new application on paper, create the associated activities using the ACT, and iteratively test the resulting application.

We then randomly selected 20 2-view chest radiographs acquired to rule out pneumonia, drawn from all such examinations performed at a 450-bed quaternary teaching hospital over a 1 week time period in 2016. The radiologist evaluated each film, in random order, in 3 sessions. During the first session, the radiologist dictated a voice-based report while viewing each x-ray, producing a text-based report. This provided a baseline report, using standard radiology evaluation and documentation methods. In the second session, the radiologist dictated while viewing each x-ray, but the radiologist’s voice was captured and encoded using the ABD prototype. In the third session, the radiologist viewed the x-ray, then used the ABD prototype’s “tap to verify” function to complete an encoded radiology template report. With both ABD voice dictation and tap to verify, the radiologist was instructed to add any pertinent additional findings that he observed in the x-ray as free text, if the ABD approach did not capture it.

We measured the comparative time needed to complete chest x-ray evaluations; the proportion of ABD-based results captured as computable data; and the completeness of the ABD-associated reports, as compared to the baseline radiology report. We assessed “completeness” by listing the specific findings found within each x-ray by each method, then the radiologist classified them as “major”—essential to the final report’s conclusions—or “minor”—items that would not change clinical recommendations.

## Results

The radiologist spent 1 hour and 45 minutes preparing an initial, paper-based draft of the application. He spent 2 hours building a working application, using the ACT. He spent 1 hour iteratively testing, modifying, and tuning the application.

The radiologist documented a total of 357 clinical findings, using any method, across the 20 randomly-selected chest x-rays that he evaluated. Finding counts ranged from a low of 8, to a high of 30 findings per x-ray.

Table 1 shows results across the 3 documentation approaches.

**Table 1:** Comparison of ABD via voice dictation and ABD via “tap to verify” manual data entry, against evaluation and documentation using traditional methods.

	Baseline	ABD – Voice recognition	ABD – tap to verify

**Time to complete an evaluation**, in seconds (95% CI)	34.8 (31.0, 38.7)	35.1 (27.7, 42.4)	29.1 (22.0, 36.2)
Proportion of 357 critical clinical findings captured as coded data	0	353 (98.88%)	353 (98.88%)
**Findings missed**			
Major	–	0	0
Minor	–	7	5
**Additional findings identified**			
Major	–	1	1
Minor	–	7	5
**Conflicting results**			
Major	–	1	0
Minor	–	11	5
**Pertinent negatives**			
Missed	–	36	34
Added	–	1	0
Proportion of 397 total clinical findings, including pertinent negatives, captured as coded data	0	354 (89.17%)	355 (89.42%)

The time required to evaluate and document a chest x-ray were similar for all 3 methods. While “tap to verify” had the lowest evaluation and documentation times, it was not significantly different from the other 2 methods.

Both ABD approaches—voice recognition with NLP and the “tap to verify” template approach—captured 353 of 357 findings (98.9 percent) as computable data (CEMs).

Neither ABD method missed any major finding captured in the traditional evaluation approach. In fact, each found 1 additional major finding that the radiologist overlooked when using the traditional approach. The radiologist concluded that the reports produced by any of the 3 methods were clinically equivalent—they would not have led to any differences in clinical conclusions drawn, or treatment actions taken.

There were differences in minor findings captured by traditional x-ray evaluation and the 2 ABD methods. Differences were even more pronounced with regard to pertinent negative findings that were reported. The traditional approach documented more than 30 pertinent negatives, across all 20 x-rays used in the study, that were missing in the ABD reports.

## Discussion

This evaluation suffers from a relatively small sample size, focused within a single subset of a specialized ancillary clinical service (radiology). It is not clear how our formal results would generalize to the full range of clinical situations documented within a comprehensive EMR. We did not evaluate the quality of the baseline radiology reports themselves. We don’t know whether different radiologists would have produced contradictory findings. This applies particularly to minor findings and to pertinent negative findings, where differences across radiologists might more likely exist.

We have used the ACT to build and test applications in a wide range of other clinical services. Those include 2 applications targeting physicians working in an Emergency Department (evaluation of a patient presenting with acute low back pain, and a full CPM for acute ischemic stroke); physical therapy (choice of therapy for patients presenting with acute low back pain); nursing (documentation of placement of IV lines, completed through voice capture and NLP translation as the nurse performs the procedure under sterile technique; evaluation of a newborn child in labor and delivery; and 12 activities associated with initial patient admission to a hospital floor); and other major radiologic procedures (pediatric abdominal x-rays, a suite of applications that cover all aspects of screening mammography and lung cancer screening). Our subjective experience in these areas is similar to what we found within the formal evaluation presented above. The method appears to be much more clinically natural for physicians, nurses, and therapists. Our ART prototype makes it is possible to document in parallel with clinical evaluation and treatment, which may greatly reduce the burden that EMRs presently place upon clinicians. One practicing physician, for example, upon using it, compared it to an “automated clinical scribe.” To the extent that it can deploy CPMs, an ABD system can anticipate clinicians’ needs and “slap the right instrument into a clinician’s hand, at the instant they need it.” We judge that it represents an optimal set of tools to deploy CDS into clinical workflows using CPM methods, where we have long-standing, deep experience.

We believe that the lower rate of pertinent negative findings captured by the ABD approach represents a flaw in our original clinical application design. Within the ACT’s content creation structure, adding templates of pertinent negatives to the existing chest x-ray module would be straightforward. In addition to capturing more complete information, such a structured list could help remind radiologists to assess and report more completely. Because our design places content generation and control directly in the hands of clinicians, we anticipate that as clinicians build and test ABD-based applications in real world clinical environments, they will iteratively improve clinical content. Applications will evolve to include most, if not all, needed documentation.

In building our initial ABD prototypes, we found a series of unanticipated secondary benefits. For example, our initial attempts to integrate full, granular activity-based costing into the ABD system have proven fruitful. It may be possible to generate very high quality financial data from clinical documentation, that can directly drive billing systems. Because activities are based on CEMs and their associated ontologies, it may be possible to automate chart abstraction, producing much more granular coding while reducing costs, dropping error rates, and shortening reporting times. ABD may enhance our ability to integrate regulatory reporting into clinical documentation. It could also produce objective staffing models, by identifying the set of activities all patients on a hospital floor will need during a work shift, then assigning the right number of the right level of health professionals to meet that need.

An ability to capture clinical documentation in a computable form has far more important implications. For example, we have tested, on a limited scale, automation of critical event reporting which presently relies primarily on fallible human beings, and often shows unacceptably low completion rates. Currently, about 10 to 20 percent of all initial diagnoses made in inpatient or outpatient settings are eventually found to be incorrect [14]. Computable data would make existing differential diagnosis systems practical on a broad scale. That could dramatically reduce mistreatment rates and greatly improve clinical outcomes. The reach and impact of clinical decision support, integrated directly into care delivery, could expand dramatically. Integrated clinical research, built into learning health care systems [13], could produce new clinical knowledge much more quickly, with deeper reach, than is presently possible. The standards-based semantic data representation that underlies computable data could greatly advance interoperable clinical data transfer. Perhaps most important, computable data would greatly increase transparency in clinical practice. Clinicians would have a far better picture of their clinical results, which would enhance their ability to advise patients as those patients make treatment choices.

## Conclusions

Activity-based design represents a confluence of “state of the art” clinical decision support methods with modern digital automation technology. It points a way toward a new future. Current electronic medical systems will need to change dramatically, to take advantage of these and other new developments. While it is not possible to say exactly what form those new systems will take, it is clear that tomorrow’s digital health care environment will be dramatically different from, and significantly better than, those that are available to support health care professionals today.
